# Navy, Army, Air and Colonial Services

**Published:** 1919-09-06

**Authors:** 


### NAVY, ARMY, AIR AND COLONIAL SERVICES.

### ROYAL NAVAL MEDICAL SERVICE.

*Qualification.*—1. Every candidate for admission into the Medical Department of the Royal Navy must be not under 21 nor over 28 years of age on the day of the commencement of the competitive examination. He must produce an extract from the register of the date of his birth, or, in default, a declaration made before a magistrate stating the date of birth.

2. He must declare: (1) His age, and date and place of birth; (2) that he is of pure European descent, and the son of natural-born British subjects; (3) that he labours under no mental or constitutional disease or weakness, nor any other imperfection or disability which may interfere with the most efficient discharge of the duties of a medical officer in any climate; (4) that he is ready to engage for general service at home or abroad if required; (5) whether he holds, or has held, any commission or appointment in the Public Services; (6) that he is registered under the Medical Act, giving the date of his registration as a medical student or of his beginning professional study; (7) whether he has been previously examined for entry into the Naval Service, and, if so, when.

3. The certificates of registration and birth must accompany the declaration, which is to be filled up and returned as soon as possible, addressed to the Director-General, Medical Department, Admiralty, S.W., before the candidate can be allowed to compete for an existing vacancy.

4. The candidate will be examined as to his physical fitness by a Board of Naval Medical Officers.

5. Candidates who have served in the Officers' Training Corps will be credited with additional marks at the entrance examination in the following proportion: Those in possession of Certificate A, 1 per cent.; those in possession of Certificates A and B, 2 per cent. of the maximum number of marks allotted.

*Examination.*—1. Entrance examination held twice yearly in London in the following subjects: (*a*) Medicine, including medical pathology and therapeutics; (*b*) surgery, including surgical pathology and clinical surgery.

No candidate will be considered eligible unless he obtains 50 per cent. of the marks in each subject.

Successful candidates will be given appointments as acting-surgeon-lieutenant in the Royal Navy, and will then enter upon a six months' course of training.

The first two months will be passed at the Naval Medical School, Greenwich.

The remaining four months will be passed at Haslar, and will be devoted to the study of naval hygiene, recruiting, physical training, diving and submarine work, radiography, anæsthetics, dentistry, etc.

*Promotions.*—Promotion to surgeon-lieutenant-commander and surgeon-commander takes place at the end of six and twelve years respectively—provided the qualifying examination for surgeon-lieutenant-commander has been passed. In addition, special promotion will be made at their Lordships' discretion as a reward for distinguished service or conspicuous professional merit.

Accelerated promotion will be granted to officers who at the qualifying examination for surgeon-lieutenant-commander obtain at least 75 per cent. of the total marks.

Promotion to the ranks of surgeon-captain and surgeon-rear-admiral are made by selection from the ranks of surgeon-commanders and surgeon-captains respectively.

The following improvements in the rates of pay of naval medical officers have been approved: Surgeon-lieutenant., on entry, 24s. a day; after six years he becomes surgeon-lieutenant-commander, with 35s. a day; and six years later becomes surgeon-commander at 45s. A surgeon-captain draws £3 5s., and surgeon-rear-admiral £5 5s., while the pay of the Medical Director-General will be £2,500 a year.

*Withdrawals.*—Officers will be allowed to withdraw, at the discretion of their Lordships, with gratuities on the following scale: After four years' service, £500; after eight years' service £1,000; after twelve years' service, £1,500; and after sixteen years' service, £2,250. There are special arrangements in respect of temporary commissions.

### ROYAL ARMY MEDICAL CORPS.

A candidate for a commission in the Royal Army Medical Corps must fulfil certain physical and medical requirements, and must be 21 years and not over 28 years of age at the date of the commencement of the Entrance examination. He must be registered under the Medical Acts in force in the United Kingdom. Before being permitted to proceed to the Entrance examination the candidate is required to attend at the War Office about two days before the commencement for the purpose of being interviewed and undergoing physical examination. The Entrance examination is held twice a year in London—January and July—a fee of £1 being charged for admission thereto. It lasts about three days. The examination is of a clinical, written, practical, and oral character. It consists of: A commentary on a case in medicine; an essay in surgery; examination and report on a clinical medical case; examination and report on clinical surgical cases; orals in clinical medicine, clinical surgery, and in medical and surgical pathology; also surgical operations and bandaging (including surgical instruments and appliances). A candidate who is successful at the Entrance examination is appointed a lieutenant (on probation), and is then required to attend at the Royal Army Medical College, London, and afterwards at the Royal Army Medical Corps School of Instruction, for the purpose of qualifying in the following subjects before his commission can be confirmed:—Royal Army Medical College: Military surgery, tropical medicine, hygiene, pathology, and organisation of military hospitals, and principles governing medical charge of troops. Royal Army Medical Corps School of Instruction: Regimental duties, drill and field training.

Further particulars relating to marks given for service in the O.T.C. or T.F., etc., and regarding the holding of civil appointments after having passed the Entrance examination are contained in "Regulations for Admission to the R.A.M.C.," which can be obtained on application to the Secretary, War Office.

A new scale of pay of officers of the Royal Army Medical Corps is shortly expected.

### ROYAL AIR FORCE MEDICAL SERVICE.

THE Medical Service of the Royal Air Force has not yet been placed upon a permanent basis; the definite conditions of service are not therefore available for publication. It is understood, however, that the general organisation of the Service will be on the lines followed by the other branches of the Force, and that the establishment will consist partly of permanent and partly of temporary officers.

Temporary officers will be required to engage for a period of four years, and may be called upon to spend part of their service at overseas stations—chiefly in Egypt or India—and must be physically fit for service in all climates.

Unless taken in for some specific duty on account of special professional qualifications, medical officers will be required to pass the usual medical tests required of other officers of the Flying Service, and at the time of entry must sign a declaration that they are willing to fly when called upon to do so.

Vacancies in the establishment of permanent commissioned officers will be filled from the temporary list by selection. Those selected for permanent commissions will count the period of their temporary service towards eventual pension; the remainder will receive a gratuity on leaving the Service at the expiration of their contract.

There will be no competitive examination on entry. Candidates must be under twenty-eight years of age, be nominated by the Dean of a recognised medical school or teaching hospital, and will be interviewed personally by the Director of Medical Services, Royal Air Force, before acceptance.

Arrangements are being made to allow of post-graduate courses after selection to permanent commission.

Promotion to the higher ranks will be by selection only.

The rates of pay and pension are not yet finally fixed, but it is understood that in the junior ranks the rates will be rather higher than the corresponding rates in the R.A.M.C. to cover the flying risk.

### INDIAN MEDICAL SERVICE.

1. Since the open competitive examination held in July 1915 for admission to the Indian Medical Service no similar examination has been held during the war, but such appointments as are required to meet the absolutely indispensable needs of the Service have been made by nomination by the Secretary of State. To assist him in making these appointments, which are limited in number to the absolutely indispensable needs of the Service, the Secretary of State for India has appointed a Selection Committee, who will summon and interview such applicants as may appear to be *prima facie* suitable, and make recommendations for appointment.

Applications for appointment should be addressed to the Secretary, Military Department, India Office, Whitehall, London, S.W. 1, and should contain concise particulars of the applicant's medical degrees and career. Applicants must be over twenty-one and under thirty-two years of age at the time of application. Particulars regarding pay, promotion, etc., in the Service can be obtained from the Military Department, India Office.

2. *Procedure for appointment in force at the last Competitive Examination.*—(1) The Regulations are those in force at the present time. They are subject to any alterations that may be determined on; (2) Every candidate must be a British subject of European or East Indian descent, and his father must at the time of the candidate's birth have been either a British subject born within His Majesty's allegiance, or a person to whom a certificate of naturalisation had been granted, or a subject of a State in India under the suzerainty of His Majesty; and such father must still be, or have continued to be till his death, a British subject or a subject of such a State in India.

Every candidate must also be of sound bodily health, and, in the opinion of the Secretary of State for India in Council, in all respects suitable to hold a commission in the Indian Medical Service. He may be married or unmarried. He must possess a qualification registrable in Great Britain and Ireland under the Medical Acts in force at the time of his appointment.

No candidate will be permitted to compete more than three times. Candidates must be between 21 and 28 years of age on the first day of the month following that in which the examination is held. But see note 1 above.

The physical fitness of each candidate will be determined by a Board of Medical Officers, who are required to certify that his vision is sufficiently good to enable him to pass the tests laid down by the Regulations. The candidate will be examined by the Examining Board in the following subjects: (1) Medicine, including therapeutics; (2) surgery, including diseases of the eye; (3) applied anatomy and physiology; (4) pathology and bacteriology; (5) midwifery and diseases of women and children; (6) materia medica, pharmacology, and toxicology.

After passing the competitive examination, the successful candidates will be required to attend a course of practical instruction at the Army Medical School and elsewhere, as may be decided, in: (1) hygiene; (2) military and tropical medicine; (3) military surgery; (4) pathology of diseases and injuries incidental to military and tropical service. This course will be of not less than four months' duration, and at its conclusion

(*Continued on p.* xviii.)

Seamen's Hospital Society in London, and during the winter the school will be moved to town in connection with the Hospital for Tropical Diseases, Endsleigh Gardens, Euston Road, N.W. The school dinner will be held at Prince's Restaurant, Piccadilly, on Wednesday, October 8, Colonel A. Balfour, C.B., C.M.G., M.D., an old student of the school, in the chair. Particulars may be obtained from the Dean, Sir Havelock Charles, G.C.V.O., at the India Office; from the Director at the school; or from the Secretary at the Head Office, Seamen's Hospital, Greenwich.

**Liverpool School of Tropical Medicine.**—The Liverpool School of Tropical Medicine is carried on in conjunction with the University of Liverpool and the Royal Infirmary, for the purposes of research and clinical teaching respectively. A special ward at the hospital has been set apart for the reception and treatment of tropical diseases, and facilities for investigation and study are given at the new laboratories of the school at the University. The next courses of instruction for the Diploma in Tropical Medicine, each lasting about three months, will commence on September 22, 1919, and January 5, 1920. The fee for a full course is 13 guineas, and for the D.T.M. examination 5 guineas. A short course, specially arranged for advanced instruction, is also held during June (fee 4 guineas).

**Both Cambridge University and the English Conjoint Board** hold examinations for the Diploma in Tropical Medicine. Neither of these bodies teaches Tropical Medicine; candidates derive their knowledge of the subject elsewheie, usually at either the London or the Liverpool School.

**Edinburgh Diploma of Tropical Medicine** The University of Edinburgh grants a Diploma in Tropical Medicine and Hygiene. The examinations are held in December and June. The fees for the first and any subsequent appearance are: Practical bacteriology, £1 1s.; diseases of tropical climates, £1 1s.; tropical hygiene, £1 1s.; tropical clinical medicine, £1 1s.; total, £4 4s.

### Special Hospitals and Graduate Study.

**Central London Throat and Ear Hospital, Gray's Inn Road, W.C.**—Three courses of instruction are open to practitioners attending the hospital: first, the course in methods of examination and diagnosis; second, the course of systematic instruction in the diseases of the nose, throat, and ear; and third, the operative surgery class. The course in methods of examination and diagnosis is introductory in character. It comprises four lessons of practical teaching in the actual examination of patients and in the manipulation of instruments. Clinical assistants, especially if they have not had any previous experience in the speciality, are expected to attend this class in order to become acquainted with the minutiæ of the methods of diagnosis, etc., The class is free to clinical assistants. To ordinary students and practitioners the fee is one guinea. Intending students (who can join at any time) are requested to give their names to the Dean. Attendance at this class is compulsory for those who desire to obtain the higher-grade certificate. The second course of systematic instruction in diseases is more advanced. It consists of over thirty lessons in all on pathology, diagnosis, and treatment. Minute details in operative surgery are not gone into, as this is left to the operative surgery class. Full syllabus may be had on application to the Secretary.

**Hospital for Consumption and Diseases of the Chest, Brompton, S.W.**—Students may attend this hospital at a fee of £1 1s. for one month, or £2 2s. for three months. Demonstrations are given in the wards, out-patient department, *x*-ray, and throat departments.

Lectures are given on Wednesday afternoons, and are free to students and post-graduates.

Clinical assistants are appointed to the physicians and assistant physicians.

For particulars apply to the Dean.

**Hospital for Diseases of the Throat, Golden Square, London, W.**, with which is amalgamated the London Throat Hospital, Great Portland Street.—The hospital contains sixty beds. There is an out-patient attendance of over 60,000 yearly. Clinical instruction in the diagnosis and treatment of disease is given in the out-patient department from 2 to 5 P.M. on Tuesdays, and Fridays from 6.30 to 9 P.M. From amongst the students junior clinical assistants are selected, whose duty it is to assist the member of the staff to whom they are appointed. For terms and further information apply to the Dean, Mr. Geo. W. Dawson.

**Hospital for Sick Children, Great Ormond Street, W.C.**—This school is one of the post-graduate teaching schools of the University of London, and is recognised by the Conjoint Board of England and by the Universities of London, Oxford, and Cambridge, as a place where undergraduates may spend six months of their fifth year in clinical work, or hold the appointments of clinical clerks and dressers. There are 240 beds, to which nearly 3,000 cases are admitted per annum, and in the out-patient department the average number of new cases each year exceeds 24,000. Clinical instruction is given daily in the wards, out-patient department, and operating theatre by members of the visiting staff. Fees for one month's attendance, two guineas; for three months, five guineas; perpetual ticket, ten guineas; clinical clerk's reduced fee of £1 1s. for one month. A certificate is given at the end of a three months' course. Further details and full syllabus may be obtained by application to the Dean or the Secretary of the hospital. A limited number of clerkships in the pathological department, affording facilities for theoretical and practical instruction in clinical pathology and bacteriology suitable for post-graduates and fifth-year students, are available. Fees for one month's couise, three guineas; two months' course, five guineas; three months' course, six guineas. For detailed syllabus application should be made to the Dean or the Secretary. The Dean is Mr. George Waugh, from whom all further information can be obtained, or from the Secretary at the hospital.

**The Hospital for Women, Soho Square, W.**—In connection with the out-patient department there has been for some years a well-organised clinical department. To meet the want increasingly felt by medical practitioners of a more intimate knowledge of the diseases peculiar to women, and of more extended opportunities for examining gynæcological cases, clinical assistants are appointed to the gynæcologists to out-patients. The appointments are open to qualified medical men and women.

The hospital contains sixty-seven beds. In the out-patient department 4,000 cases are treated every year, the total number of out-patient attendances being from 14,000 to 15,000. This large number affords exceptional opportunities for seeing and studying most of the varieties of the diseases of women. Clinical assistants are entitled to receive notice of all operations performed within the hospital upon giving their names and addresses to the hall porter, and every facility is afforded them by the gynæcologists in the out-patient department of examining patients and obtaining experience in diagnosis and treatment.

The number of clinical assistants appointed to each gynæcologist is, so far as possible, limited to two, though occasionally a third may be appointed. The clinical assistants attend on two afternoons a week, and it is advisable that they should take out a three months' course, but to meet the convenience of medical practitioners it is permitted to attend for a shorter period, and on one or more days a week if it can be arranged. A fee is charged at the rate of two guineas a month, for two days a week, which becomes due on joining. A certificate is given at the end of a three months' course.

The out-patients are seen daily at 1.30 P.M., except on Saturdays, when they are seen at 9.30 A.M. Any further information can be obtained by writing to the Secretary at the hospital.

**National Hospital for the Paralysed and Epileptic, Queen Square, W.C.**—The in- and out-patient practice of this hospital is open at 2 P.M. every day (except Wednesday and Saturday). Patients are seen by the physicians, the physicians for out-patients, and the assistant physicians. Three courses of lectures and clinical demonstrations are given in each year. The fee for out-patient hospital practice and lectures is five guineas for three months, seven guineas for six months, and ten guineas for a perpetual ticket; for in-patient clerking a fee of five guineas for three months or seven guineas for six months is charged. The museum is available for the prosecution of special work under the pathologist, for which a separate fee is charged. Women students attend the practice of the hospital.

All communications should be sent to the Dean of the Medical School.

**Ophthalmic Hospitals.**—Ophthalmic hospitals are the Central London Ophthalmic Hospital, Judd Street, W.C.; the Royal London Ophthalmic Hospital, City Road, E.C.; the Royal Eye Hospital, St. George's Circus, S.E.; and the Royal Westminster Ophthalmic Hospital, King William Street, W.C. In each of these the fees charged are very modest.

**Queen Charlotte's Lying-in Hospital.**—Qualified medical practitioners and medical students are admitted to the practice of this hospital. Last year (1918) there were 1,649 women delivered in the wards, about one-half being primiparæ. A large number of the cases were abnormal. During the present year the number of women delivered in the wards has been at the rate of over 1,800 per annum. In the ante-natal department 3,906 patients attended for examination and treatment. Certificates of attendance at this hospital are recognised by all Universities, Colleges, and licensing bodies. Fee for the course of four weeks, £8 8s. Students are accommodated at the new Residential College (5 Cosway Street), opposite the hospital.

This instruction will include: (1) Practical instruction in the methods of examination of pregnant women; (2) delivery of women in labour under the direct supervision of a medical officer of the hospital; (3) practical instruction in the treatment of the mother and child during the puerperium, including clinics held four times weekly by the visiting medical staff; (4) instruction in the clinical laboratory of the hospital.

Seventy-seven students (fifty-five men and twenty-two women) and ten qualified practitioners (seven men and three women) attended the practice of the hospital during 1918.

**Royal Hospital for Diseases of the Chest, City Road, E.C.**—This hospital, which has lately undergone extensive reorganisation, provides an excellent field for research and study in diseases of the chest, more especially in tuberculosis in its various departments. The departments in which study can be pursued comprise the in-patient, out-patient, Röntgen-ray, cardiological, pathological and bacteriological, ear, nose and throat, and dental departments. An extensive department for the prevention of consumption (tuberculosis dispensary) has been established for nearly four years. As this works in connection with three large boroughs—namely, Shoreditch, Finsbury, and Islington—a splendid opportunity presents itself for medical practitioners and students who desire to obtain an intimate knowledge of this work. The bacteriological and pathological research laboratories, under the new director, Dr. Carnegie Dickson, should provide excellent opportunities for acquiring a knowledge of bacteriological methods, and also a field for research for those who wish to pursue such studies. The cardiological department, which has recently been established, has been equipped with all the latest apparatus for the investigation of diseases of the heart. In connection with the Röntgen-ray department, there are two radiologists, who attend the hospital four days weekly. Attached to the hospital is a medical school for instruction in diseases of the chest, more especially tuberculosis, at which courses of instruction are given from time to time. There is also a tuberculosis school for nurses, where regular theoretical and thoroughly practical courses of instruction are given, for which a certificate is granted. Owing to several of the staff being engaged on "war work," it has been found impossible to arrange a theoretical course of lectures, but the clinical practice of the hospital will be open to medical practitioners and students as usual. The fees for attending the clinical practice of the hospital are as follows: One month, one guinea; three months, three guineas; six months, five guineas; perpetual ticket, six guineas. Any further information will be gladly given by the Dean, Major Barty King.

**St. John's Hospital for Diseases of the Skin.**—Out-patient Department, 49 Leicester Square, W.C. 2; In-patient Department, 262 Uxbridge Road, W. This hospital has a well-equipped in-patient department, with 40 beds. It has a School of Dermatology at 49 Leicester Square, which is conducted by the medical staff of the hospital. During the winter the free course of Chesterfield lectures is given by Dr. Morgan Dockrell, and is always well attended by the profession. Other free courses are given by Capt. W. Griffith, Dr. J. L. Bunch, and Dr. Knowsley Sibley. The out-patient department contains a spacious laboratory and an electrical department. Special courses in the pathology and bacteriology of the skin may be arranged for. The daily clinics at 2, and every evening (except Saturday) at 6, are open to practitioners, upon signing the Visitors' Book. Venereal diseases, under the Government scheme, are seen at all clinics.

